# Spindle Cell Carcinoma in the Breast: A Case Report

**DOI:** 10.7759/cureus.25702

**Published:** 2022-06-06

**Authors:** Faraz Ayyaz

**Affiliations:** 1 General Surgery, North Manchester General Hospital, Manchester, GBR; 2 General Surgery, Services Hospital Lahore, Lahore, PAK

**Keywords:** breast and endocrine surgery, plastic and reconstructive surgery, general surgery and breast cancer, spindle cell sarcoma, phyllodes tumour

## Abstract

We aim to report on a patient presenting with a rare breast tumor. The tumor was recurrent with the patient having undergone wide local excision twice previously and the diagnosis was inconclusive both times. Under our care, the patient underwent a modified radical mastectomy with a level III dissection and a transverse rectus abdominis myocutaneous (TRAM) flap for reconstruction. The entire procedure was performed in front of a live audience comprising medical students, junior doctors, and consultants, managed with audio-visual feedback enabling a real-time discussion. The final histopathological report showed a spindle-cell tumor of the breast but also mentioned that a Phyllodes tumor could not be ruled out.

## Introduction

Spindle cell carcinoma of the breast is a known but rare condition. Previous studies have shown that it comprises a total of 0.2% of all breast lesions [1,2]. Microscopically, it contains dominant spindle shape cells together with in situ /or ductal, lobular, squamous, or mixed infiltrating carcinoma [3]. Thus, the mixed morphological patterns on histopathological reports often make for a difficult diagnosis. Prognosis in spindle cell carcinoma has been reported as comparable to that of common breast carcinoma [4]. It has been found that the tissue usually contains a well-circumscribed boundary with a low incidence of lymph node metastasis. The mainstay of treatment for this disease has been traditionally a mastectomy [5].

This article was previously presented at the 18th Biennial SurgiCon on October 9-10, 2020.

## Case presentation

A 45-year-old female presented in the Surgery Outpatient Department of Services Hospital Lahore, Pakistan, with a massive, recurrent lump in the right breast which had been rapidly growing for four months prior to presentation. The patient had a BMI > 30, and no other co-morbidities were noted at the time of presentation to the hospital.

The examination findings at the time of presentation were: a large 35cm x 40cm, irregular, mildly tender mass in the right breast which was not fixed to the underlying skin. It was palpable in all quadrants and a large lymph node was palpable in the ipsilateral axilla. A 5cm x 5cm ulcerated lesion was noted on the upper, inner quadrant and a scar mark from previous surgeries was visible. There was no nipple discharge, inversion, or sinuses. There was no obvious deformity in the contralateral breast. 

The patient had undergone wide local excision of lumps in the same region twice previously within a span of 14 months prior to presentation in our hospital with differing histopathological reports. The first excision biopsy was done at a local hospital in the periphery and reported only stromal fibrosis and dilated acini and ducts with no evidence of malignancy. The mass recurred and the patient underwent a second excision biopsy four months later which came back with a report of a benign spindle cell lesion. The mass recurred once again and this time, larger and more massive than ever before and the patient decided to report to our tertiary care hospital. Upon admission, a Tru-cut biopsy was performed as a bed-side technique which came back with a report of a spindle cell lesion with evidence of fibrocystic lesions.

Procedure

The General Surgery team at Services Hospital Lahore performed a modified radical mastectomy with level III dissection. The decision to perform a level III dissection was taken due to the ambiguous diagnosis of the tumor as noted previously and the high propensity for recurrence. A level III dissection of the lymph nodes would give the patient a better chance for survival as it would completely clear out the breast tissue.

**Figure 1:**
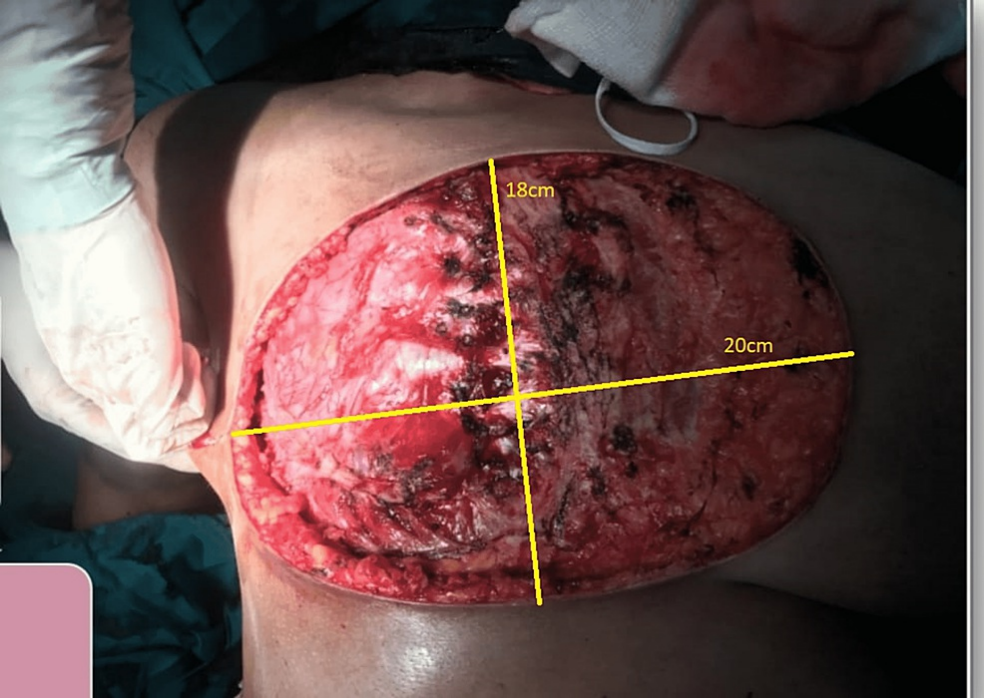
Cavity after tumor tissue excised after mastectomy

**Figure 2:**
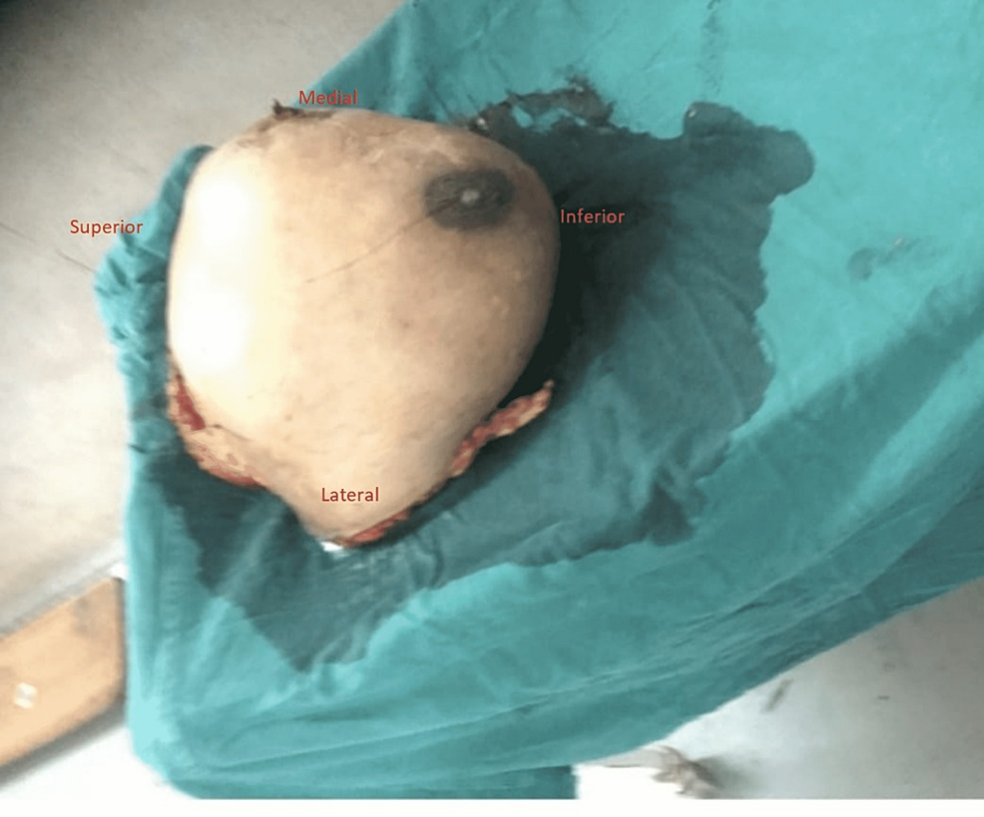
Excised breast tissue

Markings were made for the patient’s massive breast tissue under anesthesia and the subsequent dissection and excision took two and a half hours. The cavity left after excision and the tissue removed are shown below in Figures 1, 2.

**Figure 3:**
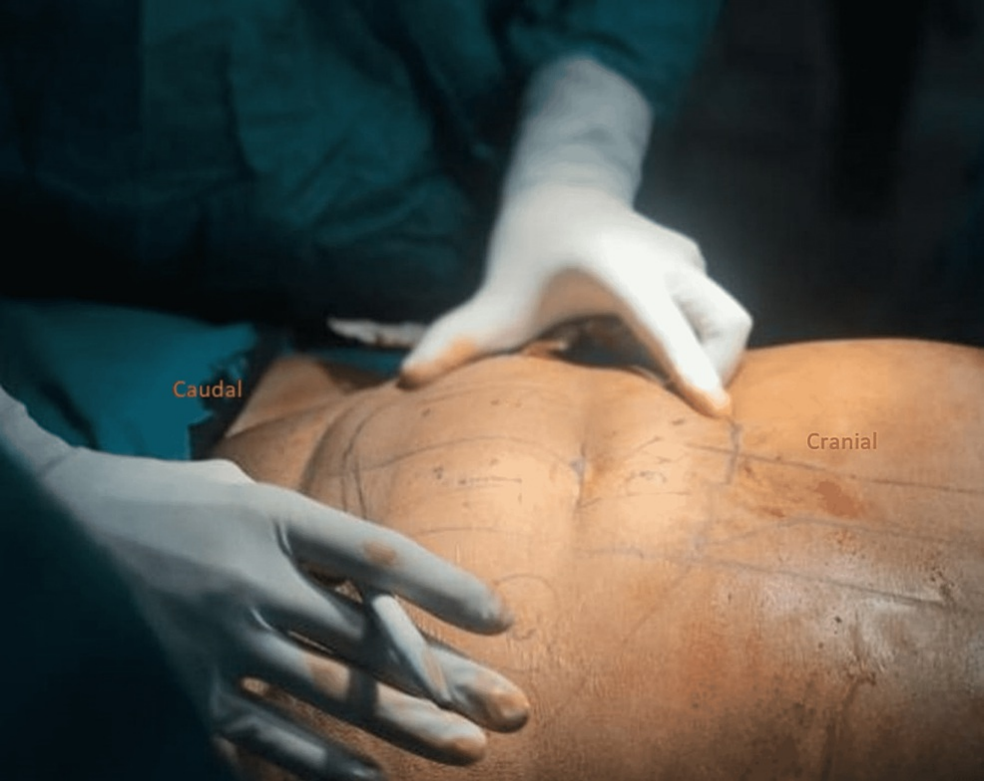
Markings for the tansverse rectus abdominis myocutaneous flap

**Figure 4:**
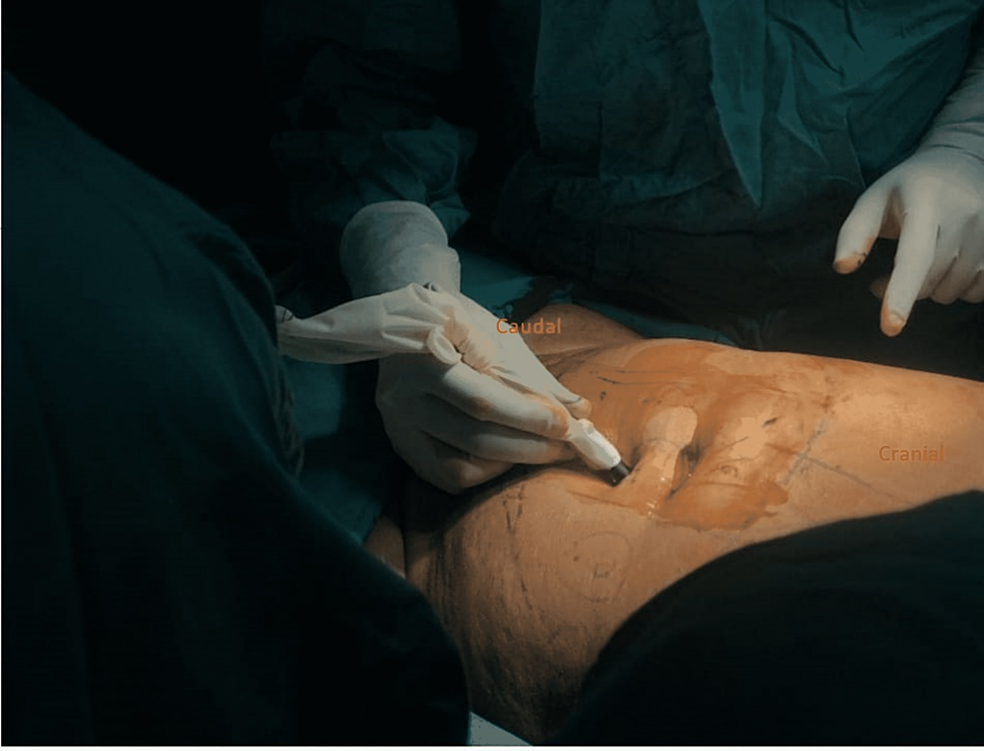
Identification of inferior epigastric artery with Doppler

**Figure 5:**
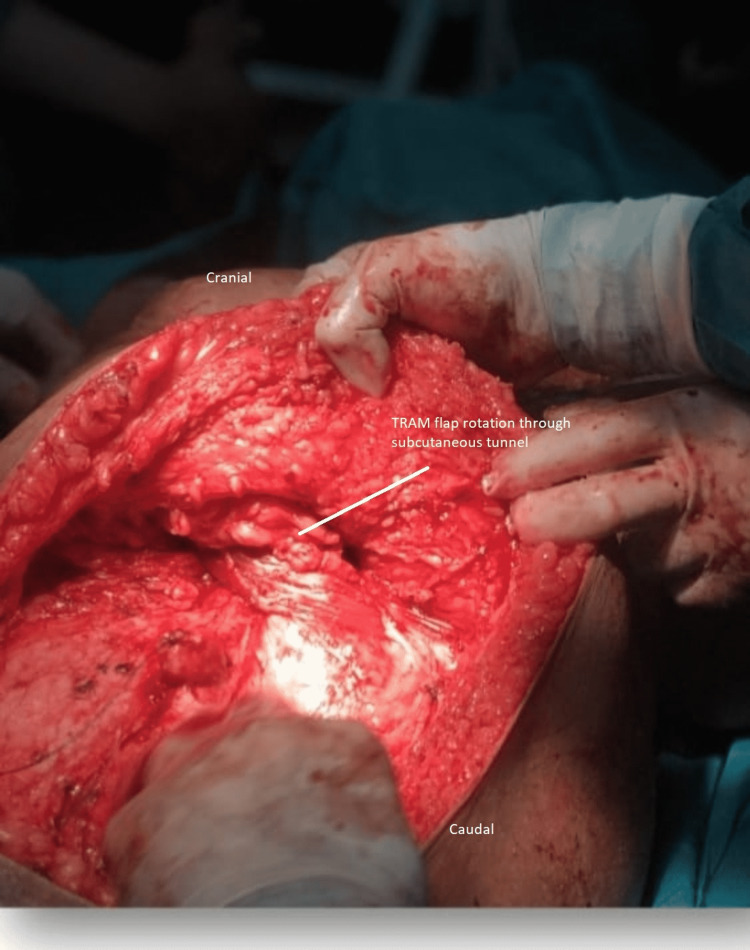
Rotation of flap through the subcutaneous tissue from the abdomen to the chest wall

**Figure 6:**
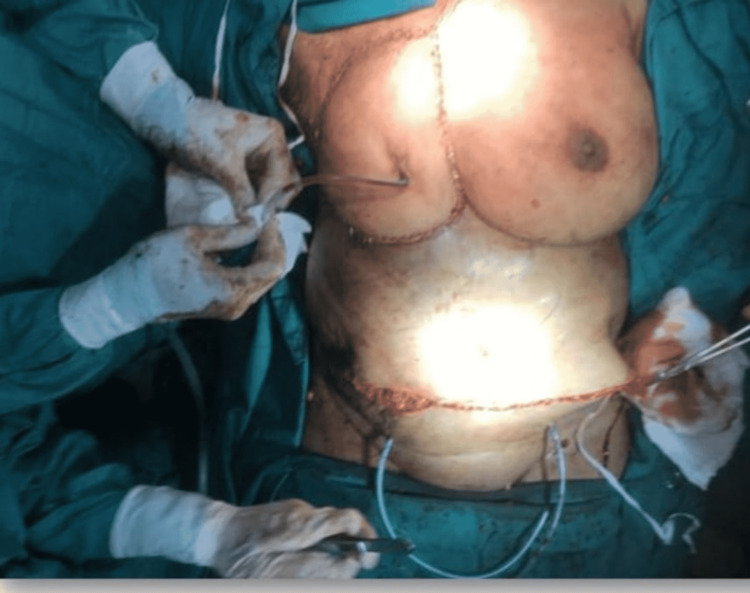
Flap placed and closure of the defect

After that, the Plastic Surgery team took over and the large 20cm x 18cm defect was noted. Specific markings were made for the transverse rectus abdominis myocutaneous (TRAM) flap were made in line and parallel to the umbilicus (Figure 3). The vessels for the flap were identified with Doppler intra-operatively (Figure 4). The flap was rotated beneath the skin and subcutaneous tissue and placed on the defect (Figure 5), filling it completely and subsequently stapled. The abdominal defect was closed and a drain was placed for drainage and the total reconstruction was a three-hour process (Figure 6).

Post-operatively, the patient was nursed in the Surgical ICU to aid post-operative recovery since the operating time for the combined procedures was over four hours. The patient was shifted to the Plastic Surgery ward after two days. The total hospital stay for the patient was three weeks. The long hospital stay was due to patient preference since she lived in a remote village and due to social reasons, follow-up for graft take-up would have been challenging for her. She remained stable throughout the stay. The flap remained viable and showed no signs of deterioration. The patient was discharged from the hospital and asked to follow up at her leisure for nipple-areola reconstruction.

**Figure 7:**
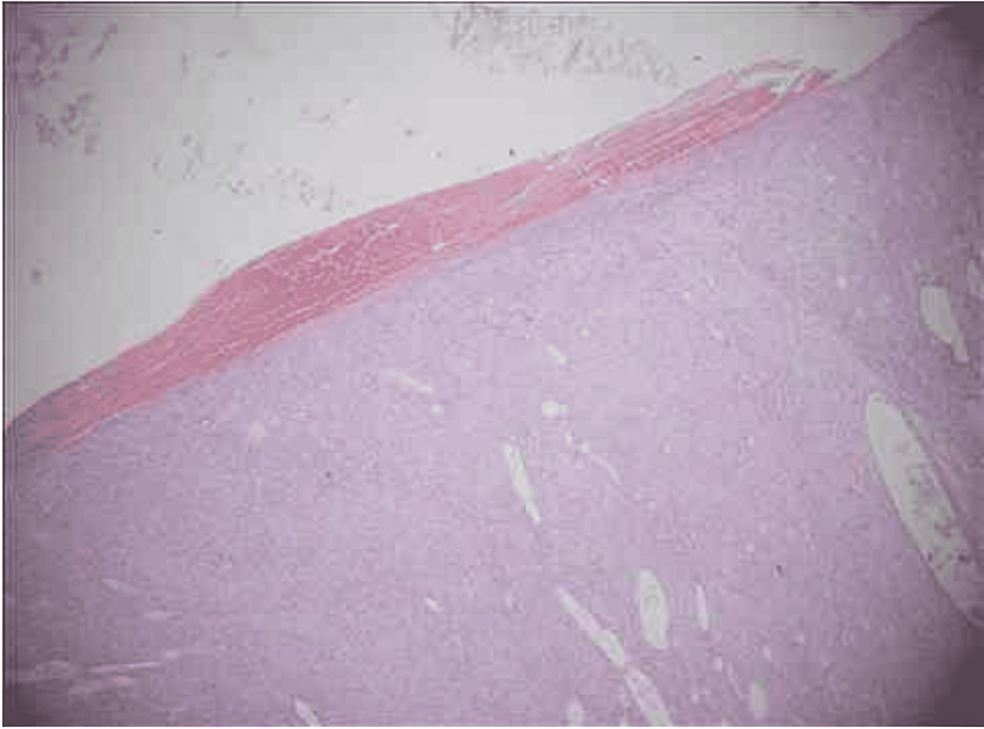
Breast tissue showing spindle cell proliferation with hypo and hypercellular areas arranged in herring bone and fascicular patterns, hyalinizing stroma

The specimen was first sent to the Pathology Department of Services Institute of Medical Sciences and came back with a histological report revealing spindle cell proliferation with hypo and hypercellular areas arranged in herring bone and fascicular patterns against hyalinizing stroma as shown in Figure 7. Pleomorphism and a mitotic count of 20 per 10 HPF was noted. The tumor was 7cm away from the superior resection margin, 6cm away from the inferior resection margin, 2cm away from the lateral resection margin and 3cm away from the medial resection margin. The deep margin was grossly involved with the tumor. Upon discussion with the pathology team, they suggested staining as well.

**Figure 8:**
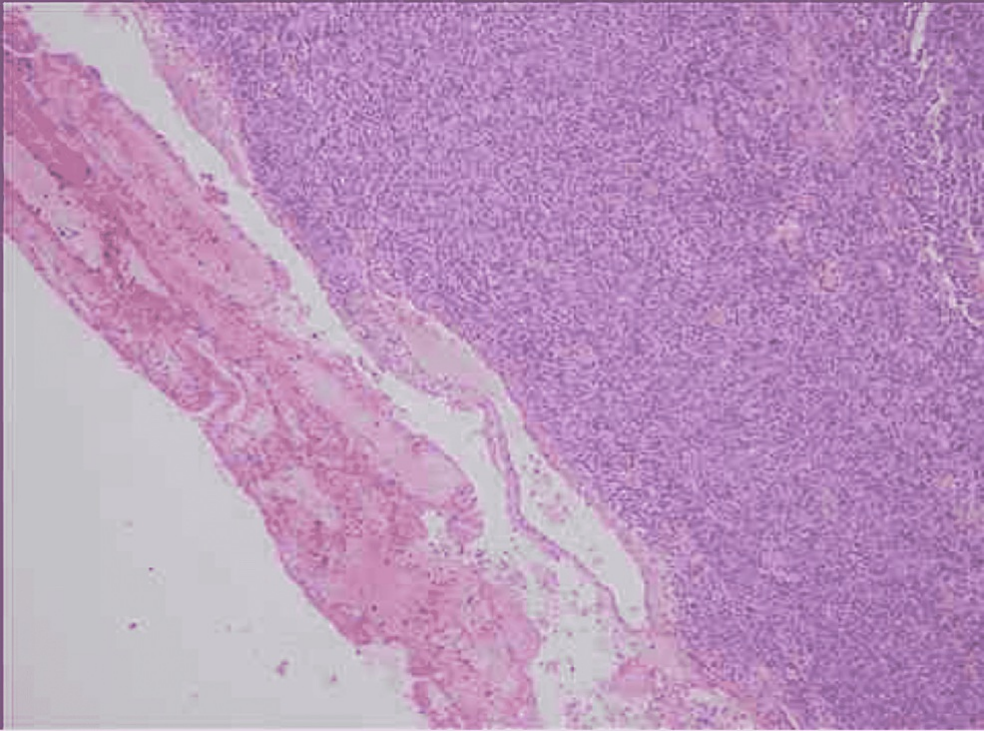
Breast tissue with hyperchromatic nuclei, spindle cells, and pleomorphism

The specimen was then sent to Chugtai Laboratory, which is a private institution and after an extensive immune-histochemistry workup, the report came back suggestive of a neoplasm showing spindle-shaped cells with nuclear enlargement, pleomorphism, and hyperchromasia. The stains used were SMA, desmin, cytokeratin, S-100, BCL-2, EMA, CK-5/6, and Caldesmon all of which were negative. A note on the report suggested, however, that the possibility of a malignant phyllodes tumor could not be ruled out. The histopathological slide is shown below in Figure 8.

## Discussion

The modified radical mastectomy with level III dissection and subsequent TRAM flap was done in front of a live audience with many senior consultants in attendance. It was managed by audio-visual feedback from the operation theatre with microphone headsets applied to the leading surgeons and two cameramen in the operation theatre. The diagnosis of the tumor was hotly debated among many peers. The underlying discussion was that spindle cell tumors rarely occur in the breast. Many of the audience suggested that the case is most likely a misdiagnosed malignant phyllodes tumor since they present with a similar history (a rapidly growing, solitary, ill-defined tumor with a tendency to occur in the breast).

 It reported a spindle cell lesion but stated that a phyllodes tumor could not be ruled out as mentioned previously. This, again, started a debate among many surgical consultants and pathologists who considered the presentation of the case with the appearance of the tumor and were divided in their diagnosis; therefore, a conclusive diagnosis could still not be reached.

Owing to the considerable confusion regarding the diagnosis of this particular case even after the previously mentioned biopsy reports, we looked at other reports of this disease around the world. Common features from all of the reports we have noted include the rarity of the disease, a challenging diagnosis owing to overlapping morphology with other tumors, a high propensity of local recurrence, and tumors themselves being locally aggressive [6-9]. We note that in our case there were similar features; the tumor had been excised twice and grew back in the course of a few months each time. The main feature that differed from previous studies in our case was the involvement of lymph nodes. It is previously noted that spindle cell carcinomas do not usually involve lymph nodes, however, in our case, the axillary group of lymph nodes was grossly involved and enlarged, warranting a level III clearance. Although a recent report has suggested newer diagnostic biomarkers using gene amplification to identify spindle cell carcinomas [10], the biopsies taken for our patient each time proved to be inconclusive. However, we also note that after the modified radical mastectomy was performed along with a level III dissection finally, the patient did not report recurrence till at least one month after discharge after which our patient self-discontinued follow-up. The TRAM flap was an ambitious undertaking considering that the tumor still was not fully diagnosed but we are glad to report that up till the patient's last follow-up it was considerably well taken.

## Conclusions

In conclusion, the diagnostic dilemma of a spindle cell carcinoma of the breast is still a problem, although it is a rarity. However, a modified radical mastectomy with a level III dissection may be possible. Depending on the size of the cavity, a TRAM flap reconstruction can also be successful within the same procedure. More studies and reports on this phenomenon and its management would further benefit our understanding of the nature of this disease.
